# Prediction of mortality in Chinese very old people through the frailty index based on routine laboratory data

**DOI:** 10.1038/s41598-018-36569-9

**Published:** 2019-01-18

**Authors:** Qiukui Hao, Xuelian Sun, Ming Yang, Biao Dong, Birong Dong, Yuquan Wei

**Affiliations:** 1 The Center of Gerontology and Geriatrics/National Clinical Research Center for Geriatrics, West China Hospital, Sichuan University, Chengdu, China; 2 Key Laboratory of Biotherapy and Cancer Center/Collaborative Innovation Center for Biotherapy, West China Hospital, Sichuan University, Chengdu, China

## Abstract

The increased risk of death in older adults can be successfully identified through frailty index (FI), based on comprehensive geriatric assessment data and self-reported data from the accumulated deficit, although the method depending on routine laboratory data (FI-LAB) remains uncertain. In the current study, the capacity of FI-LAB in evaluating the risk of mortality in a very old Chinese community cohort was analyzed. The 90-year- and above old individuals from a Dujiangyan community in Sichuan Province, China, who had completed a health assessment at baseline (in 2005) and whose laboratory data were analyzed (n = 736) from cumulative data from the Project of Longevity and Aging. The FI-LAB data was constructed from routine laboratory data and calculated as the ratio of abnormal factors in 22 variables (including red blood cells, white blood cells, and alanine transaminase) that can be assessed through blood tests. The multivariable Cox regression was used to evaluate the effect of frailty on death. In the four-year follow-up, 53.5% of the 736 participants (age = 93.6 ± 3.4 years; 67.5% women), were reported dead. The FI-LAB mean baseline value was 0.21 (standard deviation = 0.10; range = 0 to 0.55). Frailty (after adjusting for gender, age, and other confounders) could be directly correlated with increased death risk, with a hazard ratio of 1.31 (95% confidence interval (CI): 1.07–1.61) in comparison with those without frailty among the individuals. Frailty as defined by FI-LAB, established only on routine laboratory data, indicates a significant death risk in the very old people.

## Introduction

A familiar geriatric syndrome, called frailty represents a state in which the body’s physiological reserves decrease and vulnerability to stressor events increases^1^. The prevalence of frailty varies from 4.0% to 59.1% in community-dwelling adults aged 65 and above^2^. Adverse outcomes, including falls, delirium, and disability are much more likely in frail older people. As such, frailty is an emerging health priority^1,3^. Identifying frailty in older people is very important, but its diagnostic criteria are still widely debated^4^. More than 10 diagnostic assessment tools for frailty are currently available^5^, amongst which the frailty index based on cumulative health-related deficits, is one of the more commonly used methods^1,5^.

For building an index of frailty for older people, the individuals’ health-related deficits must be counted. The deficits must be chosen according to the following principles: pertaining to the health status; no early saturation; cover a range of systems; to compare the same people, the deficits that define the frailty index must be identical; the total number of deficits should be at least 30–40^6^. Typically, the deficits are symptoms, ailments, disabilities, and other measures^6,7^. The cumulative number of deficits present in an older person, divided by the sum total of all the deficits under review is defined as the frailty index^6^. For instance, if 30 deficits are reviewed, and if an individual has three of these deficits, that frailty index would be 0.01 (3/30). Thus, the range of a frailty index is 0 to 1 with higher frailty index scores suggesting a greater frailty level^8^.

In hospitalized older people and community-dwelling, the frailty index appears to be a strong predictor of adverse clinical outcomes^9,10^ and provides a quantitative measure of frailty. However, the construction of a frailty index in a busy clinical setting is time-consuming. This can be circumvented by building a frailty index that depends on routinely collected clinical data. In a Canadian Study of Health and Aging (CSHA) cohort, Howlett and colleagues reviewed 21 laboratory variables and constructed a frailty index (FI-LAB). They found that FI-LAB could identify older adults with an increased death risk (hazard ratios 1.03; 95% confidence interval: 1.02 to 1.04)^11^. Several other studies reported that FI-LAB was feasible, valid, and closely associated with frailty indexes based on complex, self-reported data for the prediction of mortality^12–14^. However, these studies only included 35 to 89 years old Caucasian individuals, with the mean age of 60 years. At the advanced stage of life, the association between abnormal lab variables and adverse outcomes may differ in relatively young old people. For example, at midlife, metabolic syndrome correlates with lower cognitive function although it shows an inverse association in those in an advanced stage of life^15–17^. Also, our team found that the frailty index (based on geriatric assessment data without cognitive evaluation) may differ in predicting late-life mortality than in relatively younger adults^18^.

The role of FI-LAB in predicting mortality in advanced/later life has not been reported until now. As such, the relationship between FI-LAB and mortality has remained unclear regarding very old populations (90 years and above). From 2005 to 2009, we carried out a cross-sectional study on 870 adults of 90 years and above age, and obtained the mortality data in 2009. This study renders the opportunity to examine the role of FI-LAB in predicting mortality at an advanced stage of life.

## Methods

### The population under study

This study was carried out in 2005 in Dujiangyan, a town in South West China, as a part of the Project of Longevity and Aging in Dujiangyan (PLAD). In this cross-sectional study, 1115 community members who were aged 90 years and above were included, which was conducted to investigate the relationships amongst longevity, ailments that are age-related, lifestyle, environment, and other aspects. The PLAD methods have been previously described in earlier studies^19–21^. Briefly, baseline data of 870 community members who consented to participate in the study, were collected through direct interviews. Trained medical staff carried out the physical examinations, measurements of body parameters, and fasting blood samples for all the individuals who participated. Formal informed consent was obtained from all participants or their legal representatives after the study details were explained to them. The Sichuan University’s Research Ethics Committee (No. 20100325) approved the study protocols. Relevant guidelines and regulations were followed while performing all the methods. For this analysis, participants lacking mortality data (53 cases) or blood samples (81 cases) were excluded, resulting in a study sample of 736 (males: 239; females: 497).

### Construction of the frailty index based on lab variables

The frailty index did not rely on a specific variable^6^. The FI-LAB was first validated using 21 laboratory variables in addition to systolic and diastolic blood pressure in the CSHA study^11^. The FI-LAB typically requires 20 or more variables, at least 70% of which are considered lab variables for a given individual^11^. Studies validating FI-LAB has been successfully replicated in many groups of elderly individuals^12,13^. In this study, we constructed a frailty index based on 22 lab variables, which were parameters for a fasting blood sample. Variables were selected according to previous studies^11^ and available items in the PLAD study. All considered variables included counts of white blood cells, neutrophilic leukocytes, platelets, hematocrit, red blood cells, hemoglobin, the mean values of corpuscular volume, cell hemoglobin, and corpuscular hemoglobin concentrations (MCV, MCH, and MCHC, respectively), blood glucose, total and direct bilirubin (TBil and DBil, respectively), alanine transaminase (ALT), albumin (Alb), globulin (Glob), urea, creatinine (CREA), uric acid (URIC), cholesterol (CHOL), high-density and low-density lipoprotein cholesterol (HDL-C and LDL-C, respectively), and triglycerides (TG). Each variable was coded as either 1 or 0, with 1 indicating that the values exceeded the normal range or cut-offs (deficits), and 0 indicating that the values were within the normal range (Table 1)^22^. Here, the sum of all existing parameter deficits, divided by the total of all the considered parameters (here, 22) defined the FI-LAB. Theoretically, the FI-LAB is an uninterrupted score between 0 to 1 for each given individual. In this study, established FI-LAB cut-points (0.21) were employed according to the previous study conducted by Hoover and colleagues^22^.

### The data for mortality and other co-variables

We collected mortality data in 2009 for all participants excluding 48 individuals (5.5%) from relatives, or neighbors and local government records. The status of the patient: survived, or dead, and the time of death was recorded. We also collected the following information as co-variables: individual’s education (illiterate, primary, secondary, and advanced level), age, gender, and chronic disease using a general questionnaire in the PLAD study through direct interviews by volunteers who were appropriately trained. All the reported chronic ailments were diagnosed by local certified physicians.

### Statistical analysis

To explain the baseline characteristics, descriptive statistics were used. The continuous or categorical variables were described using mean values, standard deviation (SD), numbers or percentages. For continuous and categorical variables, the differences between survival and frailty status (determined by FI-LAB) were evaluated by applying the unpaired Student’s *t*-test and the chi-square test, respectively. We applied regression models of Cox proportional hazard to determine the hazard ration (HR) and its 95% confidence intervals (CI) of frailty, with a function of increased mortality represented by each parameter in FI-LAB and overall frailty status. The general covariates like gender, age, and educational levels were calibrated in an adjusted Cox regression model. We also further adjusted for other aspects of lifestyle like the smoking habit, alcohol intake, exercise, and chronic ailments such as confounding factors in the Cox regression model. The SPSS version 17.0 for Windows software package, (SPSS Inc., Chicago, IL, USA) were applied for all statistical analyses and plots. The statistically significant values were set as two-tailed P at <0.05.

## Results

### The study samples and frailty

In total, 736 participants, whose age ranged from 90 to 108 and mean age of 93.6 ± 3.4 years, were included. The percentage of females was 67.5%. The participants’ median, FI-LAB, and maximum mean scores were 0.23, 0.55, and 0.21, respectively, with a standard deviation of 0.10. The FI-Lab-99th percentile score was 0.48. The overall prevalence of frailty was 50.5% (FI-LAB ≧ 0.21; 95% CI = 46.9–54.1%). Men had significantly higher FI scores compared to that of women (0.24 ± 0.10 vs. 0.20 ± 0.10; t = 5.32, p < 0.001), and the frailty group had more men than women (63.2% vs. 44.5%, respectively, X^2^ = 22.6, p < 0.001). Subjects with frailty had significantly lower educational levels, total cholesterol (TC), and LDL-C levels but significantly higher height and serum uric acid levels. The control group had a higher proportion of exercise habit than the frailty group. Table 1 presents the attributes of subjects having or lacking frailty.

**Table 1:** Characteristics of the study population according to frailty assessed by FI-LAB.

	Frailty		P value
	No (n = 364)	Yes (n = 372)	
Age (years)	93.7 ± 3.4	93.5 ± 3.4	0.418
Female (%)	75.8	59.4	<0.001**
BMI (kg/m^2^)	19.5 ± 3.2	19.1 ± 3.7	0.087
Weight (kg)	41.2 ± 8.1	41.4 ± 8.9	0.697
Height (cm)	145.4 ± 9.7	147.6 ± 10.2	0.003**
WC (cm)	77.1 ± 9.7	77.1 ± 9.1	0.945
SBP (mmHg)	141.4 ± 22.8	138.8 ± 23.1	0.128
DBP (mmHg)	73.4 ± 12.6	72.3 ± 11.6	0.233
Education level (%)			
Illiteracy	76.9	67.9	
Primary school	20.4	29.9	
Secondary school or advanced	2.8	2.2	0.012*
Smoking (%)	39.8	47.0	0.049
Alcohol drinking (%)	26.6	25.1	0.637
Having exercise habit (%)	40.2	37.0	0.379
TG (mmol/l)	1.2 ± 0.7	1.2 ± 0.7	0.804
TC (mmol/l)	4.3 ± 0.7	4.0 ± 0.9	<0.001**
HDL-C (mmol/l)	1.6 ± 0.5	1.5 ± 0.7	0.180
LDL-C (mmol/l)	2.3 ± 0.6	2.2 ± 0.6	0.017*
SUA (μmol/l)	311.1 ± 74.9	328.7 ± 97.8	0.006**
Hypertension	9.6	10.5	0.695
Cardiovascular disease	4.9	4.6	0.811
Cerebrovascular disease	2.7	1.3	0.187
Diabetes	1.4	0.5	0.282
Respiratory disease	17.6	12.9	0.077
Digestive disease	16.8	17.7	0.724
Chronic renal disease	2.5	2.4	0.963
Osteoarthritis	28.8	29.8	0.767
Status of survival			
Surviving (%)	50.8	42.2	
Death (%)	49.2	57.8	0.019*

Data are the mean ± SD unless otherwise indicated. *P < 0.05, **P < 0.01.

Abbreviations: BMI, body mass index; HDL-C, high-density lipoprotein cholesterol; LDL-C, low-density lipoprotein cholesterol; SUA, serum uric acid; TC, total cholesterol; TG, triglycerides; WC, waist circumference; SBP, systolic blood pressure; DBP, diastolic blood pressure; SD, standard deviation.

### The study sample and mortality

The sample had 53.5% rate of 4-year mortality rate. The subjects who were dead were slightly older and frailer than those in the survival group (93.9 ± 3.4 vs. 93.3 ± 3.4, t = 2.23, p = 0.026; 0.22 ± 0.1 vs. 0.20 ± 0.1, t = 2.79, p = 0.005). The death group had a higher proportion of frailty compared to that of the survival group (36.8% vs. 29.2%, respectively, X^2^ = 4.72, p = 0.030). The survival group had a higher proportion of exercise habit than the death group (45.9% vs. 32.2%, respectively, X^2^ = 4.72, p < 0.001). The survival group had less incidences of respiratory disease than those in the death group (12.3% vs. 17.8%, X^2^ = 4.27, p = 0.039). Table 2 shows the attributes of subjects according to the status of survival.

**Table 2:** Characteristics of the study population according to status of survival.

	Status of survival		P value
	Alive (n = 342)	Death (n = 394)	
Age (years)	93.3 ± 3.4	93.9 ± 3.4	0.026*
Female (%)	69.0	66.2	0.425
BMI (kg/m^2^)	19.4 ± 3.3	19.2 ± 3.6	0.374
Weight (kg)	41.3 ± 8.1	41.2 ± 8.8	0.890
Height (cm)	146.5 ± 10.0	146.5 ± 10.0	0.940
WC (cm)	76.8 ± 9.5	77.3 ± 9.3	0.504
SBP (mmHg)	140.1 ± 22.3	140.2 ± 23.6	0.935
DBP (mmHg)	72.2 ± 11.4	73.4 ± 12.7	0.155
Education level (%)			
Illiteracy	72.4	72.3	
Primary school	25.2	25.2	
Secondary school or advanced	2.3	2.5	0.985
Smoking (%)	45.6	41.6	0.272
Alcohol drinking (%)	27.3	24.6	0.402
Having exercise habit (%)	45.9	32.2	<0.001**
Frailty (%)	29.2	36.8	0.030*
TG (mmol/l)	1.2 ± 0.7	1.2 ± 0.7	0.765
TC (mmol/l)	4.2 ± 0.8	4.1 ± 0.8	0.351
HDL-C (mmol/l)	1.6 ± 0.6	1.6 ± 0.7	0.616
LDL-C (mmol/l)	2.3 ± 0.6	2.3 ± 0.6	0.981
SUA (μmol/l)	318.3 ± 86.8	321.5 ± 88.4	0.617
Hypertension	8.8	11.2	0.281
Cardiovascular disease	5.0	4.6	0.798
Cerebrovascular disease	2.6	1.5	0.288
Diabetes	0.3	1.5	0.130
Respiratory disease	12.3	17.8	0.039*
Digestive disease	16.1	18.3	0.432
Chronic renal disease	2.0	2.8	0.514
Osteoarthritis	26.6	31.7	0.128

Data are the mean ± SD unless otherwise indicated. *P < 0.05, **P < 0.01.

Abbreviations: BMI, body mass index; HDL-C, high-density lipoprotein cholesterol; LDL-C, low-density lipoprotein cholesterol; SUA, serum uric acid; TC, total cholesterol; TG, triglycerides; WC, waist circumference; SBP, systolic blood pressure; DBP, diastolic blood pressure, SD, standard deviation.

### The frailty and mortality correlation

**Figure 1:**
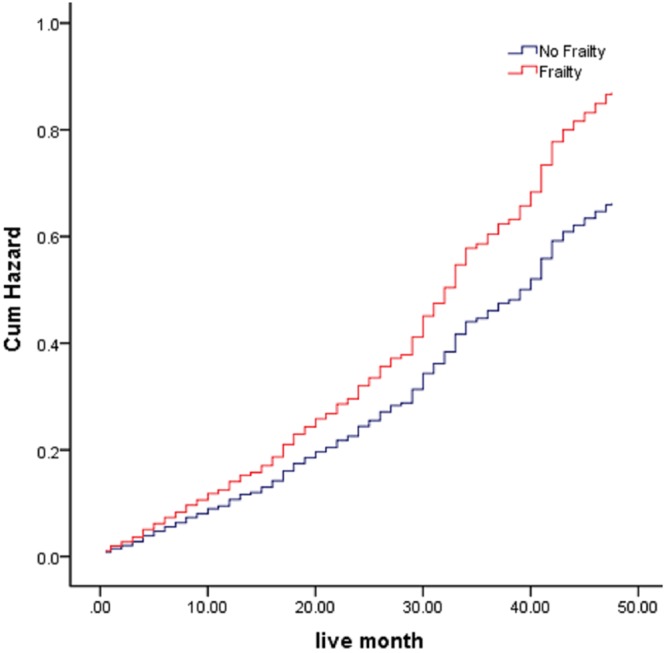
Cumulative hazard of death in the study population, according to frailty at baseline.

Statistical analysis of most variables (neutrophilic leukocytes, platelets, red blood cells, MCV, MCH, MCHC, blood glucose, TBil, DBil, ALT, Alb, Glob, CREA, URIC, CHOL, TG, HDL-C, and LDL-C) that comprised the FI-LAB did not increase the risk of four-year mortality. The hemoglobin (HR: 1.40, 95% CI: 1.05–1.86), white blood cell count (HR: 1.37, 95% CI: 1.12–1.69), and hematocrit (HR 1.26; 95% CI: 1.03–1.53) increased the risk of mortality. Table 3 shows the relationship between each selected variable in the FI-LAB and death. The outcomes from the adjusted and unadjusted Cox regression models of frailty and mortality are presented in Table 4. The frailty had a notably increased risk of mortality compared to the subjects without frailty, (HR: 1.32, 95% CI: 1.08–1.61). The model for Cox proportional hazard regression was quite stable (HR: 1.31, 95% CI: 1.07–1.61) after compensating for gender, age, alcohol intake, smoking, exercise, and several chronic ailments (hypertension, cardiovascular-, cerebrovascular-, respiratory- and digestive- diseases, osteoarthritis, chronic renal disease, and diabetes). The cumulative death hazard of the study population based on frailty at baseline is presented in Fig. 1.

**Table 3:** Routine blood laboratory variables used to construct the FI-LAB.

Standard laboratory variables	Normal range or cutoff	HR (95% CI) for 4-year mortality	P-value
White blood cells (number/L)	Men 4.0–9.2, women 3.7–9.2	1.40 (1.05–1.86)	0.021*
Neutrophil (%)	50–70	1.18 (0.94–1.48)	0.153
PLT (number/L)	100–300	1.02 (0.84–1.25)	0.822
Red blood cells (number/L)	Men 4.1–5.7 women 3.7–5.1	1.19 (0.98–1.46)	0.079
HGB	Men 131–172 women 113–151	1.37 (1.12–1.69)	0.007**
HCT	Men 0.38–0.51 women 0.34–0.45	1.26 (1.03–1.53)	0.023*
MCV	Men 83.9–99.1 women 32.6–99.1	1.01 (0.71–1.45)	0.948
MCH	Men 27.8–33.8 women 26.9–33.3	1.21 (0.92–1.58)	0.168
MCHC	Men 320–355 women 322–362	1.25 (0.98–1.58)	0.074
Blood sugar (mmol/L)	3.9–6.1	0.93 (0.76–1.13)	0.438
TC	<5.18	0.87 (0.63–1.21)	0.399
TG	<1.70	0.97 (0.72–1.29)	0.814
LDL-C	<3.37	1.15 (0.74–1.77)	0.524
HDL-C	≧1.04	0.91 (0.53–1.55)	0.720
TBIL	3.4–17.1	0.85 (0.62–1.16)	0.305
DBIL	<3.4	1.13 (0.91–1.40)	0.262
ALT	<55	0.05 (0.01–4.52)	0.191
Alb	35–55	1.54 (0.73–3.25)	0.258
Glob	9–34	1.03 (0.70–1.54)	0.868
BUN	2.9–8.2	1.14 (0.89–1.47)	0.295
Creatinine	53–140	1.24 (0.84–1.83)	0.289
SUA (μmol/l)	240–490	1.16 (0.91–1.48)	0.225

Abbreviations: HR, hazard risk; CI, confidence interval; HGB, hemoglobin; HCT, hematocrit; MCV, mean corpuscular volume; MCH, mean corpuscular hemoglobin; MCHC, mean corpuscular hemoglobin concentration; TC, total cholesterol; TG, triglycerides; LDL-C, low-density lipoprotein cholesterol; HDL-C, high-density lipoprotein cholesterol; TBil, total bilirubin; DBil, direct bilirubin; ALT, alanine transaminase; Alb: albumin; Glob, globulin; BUN, blood urea nitrogen; SUA, serum uric acid. *P < 0.05, **P < 0.01.

**Table 4:** Estimate of the accuracy of the FI-LAB on mortality, modeled with Cox regression.

	No frailty	Frailty HR (95% CI)
Unadjusted model	1 (Reference)	1.32 (1.08, 1.61)
Adjusted model 1^a^	1 (Reference)	1.33 (1.09, 1.63)
Adjusted model 2^b^	1 (Reference)	1.31 (1.07, 1.61)
Adjusted model 3^c^	1 (Reference)	1.31 (1.07, 1.61)

^a^Adjusted for age, gender, educational levels.

^b^Adjusted for age, gender, educational levels, smoking, alcohol drinking, exercise habit.

^c^Adjusted for age, gender, educational levels, smoking, alcohol drinking, exercise habit, hypertension, cardiovascular disease, cerebrovascular disease, diabetes, respiratory disease, digestive disease, chronic renal disease, and osteoarthritis.

Abbreviations: HR, hazard risk; CI, confidence interval. *P < 0.05, **P < 0.01.

## Discussion

In this study, we investigated the correlation between mortality and FI-LAB in 90–108 years old individuals in Dujiangyan city, Sichuan Province, China, to gain an understanding of how frailty is assessed based on abnormal routine blood parameters that influence the risk of death at an advanced stage of life. We believe that this is the first study of its kind that evaluates the link between FI-LAB and mortality in a specific population. We show that frailty assessed through routine blood parameters is linked to an increased risk of mortality than those of the control group, which indicates that attention must be given to abnormal routine blood parameters even among very old people.

We also found that men were more prone to frailty than women, according to both FI-LAB scores and frailty prevalence. This differed from previous studies employing the Fried phenotype and frailty index defined on the basis of comprehensive geriatric assessment^2,23–25^. The majority of studies found that females had a greater frailty burden than males, even though females usually live longer than men^26^. One reason for this phenomenon was that the frailty phenotype and frailty index are based on comprehensive geriatric assessments, which may not include all variables that affect life expectancy in older people^26^. One previous study that employed laboratory parameters to compile the frailty index also found that men had higher FI-LAB scores than women among older inpatients^14^. The study, however, included only 306 inpatients with an average age of 82.9 ± 6.4 years, with the mean FI-LAB scores of 0.34 ± 0.15, which was higher than the FI-LAB score in our sample (0.21 ± 0.10). The main reason for these differences is that older people who require hospitalization are typically frailer than those from the community. Laboratory variables were also more objective than health-related deficits from self-reported data^27^. This indicated that the FI-LAB can capture other factors that influence mortality to a higher level than other frailty assessment methods, particularly amongst old men.

Several studies have reported that the FI-LAB can predict mortality, and yielded results similar to this study. However, these studies included participants with ages ranging from 35 to 89 years old^11–14^. One of the studies found that the association of FI-LAB and mortality was not statistically significant amongst those aged 20–39 years old^12^. Thus, the role of FI-LAB in predicting death differed amongst age groups and the relationship between FI-LAB and mortality was indefinable in very old people. This, therefore, extends previous conclusions to a group with very old individuals.

Interestingly, we found that the majority of variables that made up the FI-LAB did not elevate the four-year mortality risk, except hemoglobin (HR: 1.40, 95% CI: 1.05–1.86), white blood cell count (HR: 1.37, 95% CI: 1.12–1.69), and hematocrit (HR: 1.26, 95% CI: 1.03–1.53). After excluding these variables (hemoglobin, white blood cell count, and hematocrit), we also found that an FI-LAB score (based on 19 other variables) divided by 0.25 or 0.21, could show a higher risk of mortality in Cox regression models (HR: 1.26, 95% CI: 1.01–1.57; HR: 1.17, 95% CI: 0.96–1.42, respectively). These results are in accordance with the theory of health-related deficits accumulation, developed by Rockwood and colleagues, and the accumulation of subclinical deficits was demonstrated by FI-LAB^12,28^. In clinical practice, we must also be aware of the accumulation of these abnormal variables in the laboratory parameters.

In a four-year retrospective cohort study, Cheung and colleagues included 266 patients with trauma (mean age 76.5 ± 7.8 years), collected samples within 48 hours of presentation at the hospital and constructed an FI-LAB using 23 parameters^29^. The study found that frailty on admission, as defined by an FI-LAB > 0.4, was not associated with discharge destination, in-hospital complications, and other adverse outcomes. To our knowledge, no previous studies have focused on the correlation between FI-LAB and disease prognosis in older people. Whether FI-LAB can be used for clinical decisions should be further investigated.

Numerous studies have found that low levels of education are a risk factor of frailty and associated physical and cognitive function in older people^25,30–32^. While education is not directly involved in the pathophysiology of frailty, it can benefit an individual’s health by selecting a health-related lifestyle. However, the evidence regarding the protective effects of higher education in frailty amongst very old individuals was scare. In this study, we indicated that the education found amongst nonagenarians or centenarians had protective effects. Consistent with previous studies, We also found that the risk of frailty is directly associated with exercise^33^. Moreover, the guideline of frailty management in older people also recommends exercise^34^. Concomitantly, this study supports the idea that older individuals should maintain exercise at advanced stages of life. However, we did not adjust potential variables and bias in the results may therefore exist.

Nevertheless, the study had a few limitations, so, data in this study must be explained with caution. Firstly, the number of subjects was smaller (n = 736) than the cohort studies from Canada. In addition, the subgroup analysis based on frailty at different levels was limited due to the small number of participants. However, this remains the first study to explore the role of FI-LAB in predicting mortality among very old individuals, and it is difficult to collect data in this age group. Secondly, we only included nonagenarians or centenarians (Han Chinese) which may cause survival bias, which is obvious while studying the individuals of this very old group. Thus, we cannot extend the present findings to general old people and other races in general. Thirdly, other potential confounders, including income and a family history of chronic disease were not adjusted for. The majority (90%) of participants were farmers and lived in rural areas. Many differences in the characteristics of frailty between rural and urban populations in Chinese elderly people exist^35^. Therefore, the urban population may not have been effectively represented by the participants in this study. Fourthly, this study did not provide data involving grip strength and speed of walking, which was part of the Project of Longevity and Aging in Dujiangyan, thus, the role of frailty defined as the frailty phenotype could not be explored. However, the frailty- phenotype and -index are comparable, particularly when the cut-off point of a frailty index is set at 0.20–0.25^22,36^. Furthermore, recent studies found that both the frailty index and frailty phenotype can predict the three-year mortality risk, even though the discrimination of frailty gradually declines with increasing age^37^.

## Conclusions

Frailty, as evaluated by routine blood parameters is linked with a higher risk of mortality. This indicates that more attention should be paid to abnormal routine blood parameters amongst very old individuals when the death risk at advanced stages of life is assessed.
